# Emotion regulation of others’ positive and negative emotions is related to distinct patterns of heart rate variability and situational empathy

**DOI:** 10.1371/journal.pone.0244427

**Published:** 2020-12-31

**Authors:** Josiane Jauniaux, Marie-Hélène Tessier, Sophie Regueiro, Florian Chouchou, Alexis Fortin-Côté, Philip L. Jackson

**Affiliations:** 1 École de Psychologie, Université Laval, Québec, Canada; 2 Centre Interdisciplinaire de Recherche en Réadaptation et Intégration Sociale (CIRRIS), Québec, Québec, Canada; 3 Centre de recherche CERVO, Québec, Québec, Canada; 4 IRISSE Laboratory (EA4075), UFR SHE, Université de La Réunion, Le Tampon, France; 5 Département de Psychologie, Université de Montréal, Montréal, Québec, Canada; Sapienza University of Rome, ITALY

## Abstract

Although emotion regulation has been proposed to be crucial for empathy, investigations on emotion regulation have been primarily limited to intrapersonal processes, leaving the interpersonal processes of self-regulation rather unexplored. Moreover, studies showed that emotion regulation and empathy are related with increased autonomic activation. How emotion regulation and empathy are related at the autonomic level, and more specifically during differently valenced social situations remains an open question. Healthy adults viewed a series of short videos illustrating a target who was expressing positive, negative, or no emotions during a social situation (Positive, Negative, or Neutral Social Scenes). Prior to each video, participants were instructed to reappraise their own emotions (Up-regulation, Down-regulation, or No-regulation). To assess autonomic activation, RR intervals (RRI), high frequency (HF) components of heart rate variability (HRV), and electrodermal activity phasic responses (EDRs) were calculated. Situational empathy was measured through a visual analogue scale. Participants rated how empathic they felt for a specific target. Up- and Down-regulation were related to an increase and a decrease in situational empathy and an increase in RRI and HF, respectively, compared to the control condition (No-regulation). This suggests increased activity of the parasympathetic branch during emotion regulation of situational empathic responses. Positive compared to Negative Social Scenes were associated with decreased situational empathy, in addition to a slightly but non-significantly increased HF. Altogether, this study demonstrates that emotion regulation may be associated with changes in situational empathy and autonomic responses, preferentially dominated by the parasympathetic branch and possibly reflecting an increase of regulatory processes. Furthermore, the current study provides evidence that empathy for different emotional valences is associated with distinct changes in situational empathy and autonomic responses.

## Introduction

Empathy, the ability to feel and understand others’ affective states, is deeply rooted in everyday social interactions [1]. In the field of social affective and cognitive neuroscience, theoretical models deconstruct empathy into two main interacting components, namely the affective and cognitive components [1–4]. The affective component refers to vicariously sharing others’ emotions, while the cognitive component refers to the ability to understand others’ feelings [1]. Although emotion regulation is a component in some models of empathy [1, 3], emotion regulation and empathy have been assessed separately in empirical studies [5]. Investigations on emotion regulation have been primarily limited to intrapersonal processes, leaving the interpersonal processes of self-regulation rather unexplored [6]. In the process model of emotion regulation, emotion regulation, which entails a variety of sub-types of regulatory mechanisms and strategies, is defined as the ability to shape the nature and/or to reduce (i.e. down-regulate) or promote (i.e. up-regulate) the intensity of our own emotions [7]. In the models of empathy, emotion regulation is described as a cognitive top-down process that can down-regulate [1, 3], but also up-regulate [2], the spontaneous affective reactions resulting from the observation of the emotional states of others. The function of emotion regulation is thereby to influence (i.e. to increase or decrease) the intensity of the affective component, which in turn promotes an adapted empathic response [8].

The close link between emotion regulation and empathy has been proposed in theoretical work for over two decades. For instance, Eisenberg’s and colleagues claimed that under the influence of different levels of emotion regulation capacities, individuals tend to present two main dispositional empathic responses to others’ needs, either personal distress or empathic concern [9]. Personal distress is a self-focused reaction to someone’s emotional state which leads to an aversive experience of anxiety or distress, whereas empathic concern is an altruistic motivation in response to others’ needs [10]. Low emotion regulation capacities have been proposed to be related to high emotional arousal and personal distress [11]. In contrast, high emotion regulation capacities have been proposed to be associated with moderate levels of emotional arousal and empathic concern [11]. Thereby, strong emotion regulation capacities may serve empathy by reducing the probability of experiencing others’ emotions as aversive, enhancing one’s level of concern towards another person. This would then ensure an equilibrium between affective and cognitive empathy [1] and prevent maladaptive patterns of empathy [7, 11]. Although these theoretical assumptions are intriguing, empirical data that support them remains rather scarce [8].

Measures of the autonomic nervous system (ANS) can provide important insights regarding the relation between empathy and emotion regulation [12]. Indeed, humans tend to coordinate their autonomic responses with those of others through the perception of socio-affective cues during empathy [13]. The combined activity of the sympathetic nervous system (SNS) and parasympathetic nervous system (PNS) controls the cardiac activity [14]. Through an electrocardiogram (ECG), it is possible to study autonomic cardiac activity. The interval of time between two heart beats can be calculated from one R peak to another R peak, namely the RR interval (RRI). The RRI variability, or the fluctuations in heart beats over time, serves as a measure of the autonomic state. The RRI variability indicates whether cardiac activity is being regulated, namely if autonomic cardiac activity is increasing or decreasing [15]. More specifically, spectral metrics of the HRV are widely recognized as valuable tools to index autonomic activity [16, 17]. It is usually recognized that high frequency power (HF) between 0.15 to 0.40 Hz is primarily under parasympathetic control [16, 18] and is an important tool to index parasympathetic responses during self-regulation at the emotional, cognitive, and social levels [19].

Considering the difficulties to specifically index sympathetic activity with cardiac measures [19], electro-dermal activity (EDA) is a valuable bio-signal that gives important insights into the autonomic state [20]. EDA represents the electrical variations in the skin associated with sweat gland function. Sweat gland are primarily under sympathetic control of the ANS [20] and there would be no parasympathetic innervation of eccrine sweat glands in the skin [21]. A phasic variation of the EDA, namely an EDA response (EDR), also referred to as galvanic skin responses (GSR) [21], corresponds to a brief and increase in the EDA signal usually appearing after an arousing event [22]. An EDR is considered an indicator of a local arousal process related to the sympathetic activity [21]. Indeed, increases in sympathetic activity are associated with increases in sweating, which have been related to “autonomic arousal” [22]. In psychophysiological studies, the amplitude of the EDR (EDR-amp) is often used and interpreted as an index of emotional and cognitive arousal [20–22]. Indeed, sympathetic activity is closely related to emotion, and EDR is often used as an index of emotion-related sympathetic activity [20–22]. EDR is also sensitive to low arousal changes that are cognitively determinate [21].

Preliminary data from psychophysiological studies have begun to shed some light on the relationship between emotion regulation, empathy, and autonomic responses. On one hand, studies show that an increase of the HRV is usually related to greater emotion regulation capacities [23–25], both at rest [26] and during emotional tasks [27, 28]. For instance, Fabes and Eisenberg [29] measured the emotion regulation capacities, including strategies such as emotional regulatory control, inhibitory control, and autonomic activity, through the Physiological Reactivity Questionnaire (PRQ) [30]. Greater scores on the PRQ correlated positively with greater HF [29]. Another study showed that participants who attempted to down-regulate their emotions during a conversation of an upsetting film, either by suppressing their emotions (i.e., an emotion regulation strategy of suppressing the experience of emotions) or reappraising their emotions (i.e., a cognitive change strategy of modifying how one appraises a situation in order to alter its emotional significance) [7], showed greater levels of resting HF than participant conversing naturally [27]. The RRI also increased in recovering alcoholics in response to alcohol cues, only when they later reported an increased capacity to resist a drink [26]. In sum, greater emotion regulation capacities are usually related to greater levels on the metrics of the HRV, which suggest an increase in the parasympathetic activation.

On the other hand, some studies showed that increases in the HRV and EDR are related with greater dispositional empathy, namely the consistent and stable trait of an individual to be empathic over time [31]. For instance, Lischke et al. [32] investigated if inter-individual differences in resting state HF were associated with inter-individual differences in dispositional empathy, as measured by a questionnaire (i.e. The Empathy Quotient) [33]. Results showed that individuals with higher HF compared to lower HF reported more dispositional empathy. An increase in HF was also associated with an increase in the dispositional empathic score [32]. In Chiesa and colleagues’ study [34], empathic processing was investigated during a subliminal perception task of pleasant and painful facial expressions. Pupil dilation size, which indexes sympathetic activity, and pleasantness ratings of a neutral target, were gathered during the task. Dispositional empathy was measured by the Interpersonal Reactivity Index questionnaire (IRI) [35, 36]. An increase in pupil diameter was associated with a higher score on the empathic concern subscale of the IRI during the presentation of painful compared to pleasant facial expressions [34]. In a series of four studies, links between parasympathetic activity and compassion has been shown [37], namely the feeling of sorrow or concern for the suffering of another person paired with the desire to remove suffering from others [8]. Indeed, participants presented greater HF during a task inducing compassion compared to a neutral condition and a positive emotion condition. HR and respiration rate were usually lower during compassion without any change in the EDR. The authors suggested that compassion is related with an increase of parasympathetic activity [37]. Lastly, Buffone and colleagues [38] examined how different perspectives, i.e., imaging oneself in the situation of a suffering person or merely thinking about the other’s feelings, affected cardiovascular responses, including the HR. The experiment consisted of reading a fictional description of a personal hardship and then recording a helpful video to this person while cardiovascular measures were recorded. More specifically, participants needed to briefly paraphrase the persons’ problem and respond to that problem in a manner that they found appropriate. Three groups of participants were formed for each perspective condition. Self-oriented compared to the other-oriented perspectives led to an increase physiological arousal/threat and self-reported distress. Data from developmental studies also support the notion that autonomic responses predict empathy, and more broadly prosocial behavioural [39–41]. Taking together, these findings suggest that both empathy and emotion regulation are related with changes in autonomic responses. The relation between both concepts is still however equivocal and may be limited to the autonomic level.

Diverse strategies can be used to engage emotion regulation processes [42]. It has been shown that individual differences in emotion regulation strategies combined with individual differences in dispositional empathy significantly impact individual differences in affective distress, including depression, anxiety, and stress [43]. In Powell’s survey, dispositional empathy was measured with the Questionnaire of Cognitive and Affective Empathy (QCAE) [44], and emotion regulation was measured with the Emotion Regulation Questionnaire (ERQ) [45], and affective distress was measured with the Depression, Anxiety and Stress Scales-21 (DASS-21) [46]. Results showed that a greater tendency to use reappraisal was related to less depression, anxiety, and stress. Conversely, a greater tendency to use suppression was associated with more depression, anxiety, and stress. Moreover, affective empathy predicted greater depression and anxiety when the tendency to reappraise was low, but not when the tendency to reappraise was high. Cognitive empathy predicted less anxiety and stress when suppression was low, but not when it was high. Reappraisal thus appears to lead to better psychological outcomes, at least when combined with higher dispositional affective empathy.

Although these previous studies suggest that emotion regulation and empathy are associated with greater parasympathetic activation, the equivocal nature of this evidence raises questions. Can emotion regulation impact empathic responses and parasympathetic activation? Are there any differences in empathic responses and parasympathetic activation between up- and down-regulating our emotions? In addition, these previous studies have focused mainly on dispositional empathy, while empathy has been shown to be a dynamic phenomenon that can vary among situations [47]. As Batson and colleagues [48] have already proposed, empathy is generally more situational than dispositional. Measuring empathy by a questionnaire is sensitive to social desirability and the desire to present oneself as empathic [48]. As such, some authors have suggested that measures of situational empathy, namely the immediate empathic response in a specific situation [31], can contribute to increased external validity of empathy studies [49]. Moreover, corroborating measures of situational empathy and dispositional empathy can provide a more valid portrait of one’s proclivity to be empathic. Also, previous studies have mostly examined empathy in the context of negative emotions. Empathy for positive emotions is an emerging field of research, but only a handful of studies have considered emotional valence [4]. Morelli and colleagues [4] pointed out that empathy for positive emotions occurs during many social situations and correlates positively with personal satisfaction, social well-being, and pro-social behaviours. This suggests a need for increased focus on emotional valence in empathy research.

The general aim of this work was thereby to contribute to the empirical foundation regarding how emotion regulation, and more specifically, how reappraisal, impacts empathy. By using a psychophysiological approach, the current study aims to 1) examine the effect of up-regulation and down-regulation on sympathetic and parasympathetic activation, and situational empathy, 2) compare the effect of emotional valence (positive, negative, and neutral) on sympathetic and parasympathetic activation, and situational empathy, and 3) examine the interaction effect between up- and down-regulation and emotional valence on sympathetic and parasympathetic activation and situational empathy. Healthy participants viewed videos of positively and negatively valenced scenes of social-emotional interactions. Participants were instructed to up-regulate and down-regulate their emotions, while electrodermal and cardiac activities were recorded. In order to measure situational empathy, participants rated how empathic they felt for a specific target. Based on the theoretical assumptions postulated between empathy and emotion regulation, it was expected that differences in up-regulation and down-regulation and differences in positive and negative emotional valence would be related to differences in situational empathy and sympathetic and parasympathetic activation. More precisely, it was hypothesized that down-regulation would be associated with a decrease in SNS activity and an increase in PNS activity, i.e. a decrease of the autonomic activity due to an increase of the parasympathetic influence, in addition to an increase in situational empathy, compared to a control condition. We expected that up-regulation would be associated with an increase of SNS activity and a decrease in PNS, i.e. an increase of the autonomic activity due to sympathetic influence, in addition to a decrease in situational empathy, compared to a control condition. It was further hypothesized that negative compared to positively valenced emotions would lead to an increase of sympathetic activity, a decrease of parasympathetic activity, and an increase of situational empathy.

## Materials and methods

This study was approved by the Institut de réadaptation en déficience physique de Québec (IRDPQ) ethics committee (No. 2015-410).

### Participants

Fifty-nine healthy adults were recruited through the student and staff mailing list of Laval University. None of the participants reported a history of neurological, psychiatric or pain disorders. Five participants were excluded due to technical problems during data acquisition. The final sample was composed of 26 men (*M* = 24.5 years old; *S*.*D*. = 3.4) and 28 women (*M* = 24.1 years old; *S*.*D*. = 4.0). All participants provided written informed consent prior to the study and received compensation for participating.

### Procedure

Participants completed the consent forms upon arrival to the laboratory. They were then asked to sit in a chair in a dimly lit room at a distance of 50 cm from the eyes to a 22-inch computer screen (HP Compac, LA2205wg) with a resolution of 1920 x 1080 and a refresh rate of 60 Hz. Participants then completed the experimental task, and then the questionnaires. Before leaving, participants were debriefed.

### Stimuli

To solicit empathic responses, fourteen validated videos from the Emotional Movie Database (EMDB) illustrating social interactions of individuals expressing negative or positive emotions (Negative and Positive Social Scenes) were used [50]. Negative Social Scenes illustrated a woman and/or a man in a negative situation (e.g., argument) and expressing sadness, pain, anger, or crying. Positive Social Scenes illustrated a woman and a man, or only a woman, in a positive situation (e.g., getting married). As a control, and in order to gather films presenting social interactions with very low arousal and emotional valence, additional films were taken from other datasets [51, 52]. Indeed, the EMDB only provides neutral films of the object, which does not contain social information [50], and thereby were less suitable as a control for the current research. All neutral films were validated and classified based on subjective ratings of valence and intensity dimensions according to the theoretical background of Russell’s circumplex model of emotion [53]. All neutral films elicit low intensity and neutral valence ratings [51, 52] and have previously been used as a control condition in diverse emotional tasks [50, 54, 55]. Neutral social scenes illustrated a man or woman in an office, reading a book, walking, or talking. All emotional and neutral videos were edited to forty seconds. Across all the films clips, some scenarios (*n* = 8) presented targets communicating verbally, in addition to expressing their emotions non-verbally. All videos were validated without soundtracks. More details on the videos are available in the Supplementary Material (see S1 Appendix).

### Experimental design

Prior to all videos, instructions were presented informing participants which emotion regulation strategy to adopt (i.e. Down-regulation, No-regulation, Up-regulation). Then, the image of the main target (i.e., the character the participants were instructed to focus on) was presented. For some videos, two main targets were presented; in these cases, half of the participants were asked to focus on one target and the other half were asked to focus on the other. The experimental task consisted of a 3 (Emotion Regulation: Down-regulation, No-regulation, Up-regulation) X 3 (Valence: Negative Social Scenes, Neutral Social Scenes, Positive Social Scenes) factorial-repeated measures design (Table 1). The experimental task was implemented in E-prime software (E-prime studio 2.0, Psychology Software Tool, Inc.) and was divided into three blocks corresponding to each of the emotion regulation conditions. The No-regulation condition was always presented first, as this condition aimed to capture spontaneous activity and determine the initial level of autonomic activity unprimed by any emotion regulation strategy. The Up- and Down-regulation conditions were counterbalanced across participants. Within each block, 21 trials were conducted, corresponding to 7 trials per Valence condition. Videos were presented in a pseudo-random order to ensure that no more than two videos of the same valence were presented consecutively. Each trial consisted of the following successive elements: the instruction indicating which emotion regulation strategy to adopt (1 sec.), the image of a main target that participants were to focus on (2 sec.), a fixation cross (2 sec.), and the video (40 sec.). Prior to the experimental task, three familiarization trials, one per Valence condition, were completed to ensure that the experimental task was understood.

**Table 1:** The nine experimental conditions of the task.

	Emotion Regulation		
Valence	*Down-regulation (Down)*	*No-regulation (No)*	*Up-regulation (Up)*
*Negative Social Scenes (Neg)*	Down-Neg	No-Neg	Up-Neg
*Neutral Social Scenes (Neu)*	Down-Neu	No-Neu	Up-Neu
*Positive Social Scenes (Pos)*	Down-Pos	No-Pos	Up-Pos

In order to manipulate emotion regulation, the task employed reappraisal strategies based on the emotion regulation meta-analysis of Web and colleagues [42]. The instruction for the No-regulation condition was to watch the target, with the following instruction prior to the video: “I watch this person.” For the Up-regulation condition, participants were instructed to imagine themselves in the target’s situation. Participants were specifically asked to report their own emotions and not how they thought they should feel or how they thought the target in the video was feeling. The instruction for this condition was thus: “I feel the emotions of this person.” Lastly, for the Down-regulation condition, participants had to view their emotions from a distanced/third-person perspective to introduce some distance between them and the target’s emotions. The instruction was: “I distance myself from the emotions of this person.”

### Self-report measures

#### Positive affect and negative affect scales

Considering the aims of the study and that emotion regulation capacities can be greatly influenced by mood and affects [6, 56], the French-Canadian translated version [57] of the *Positive Affect* (PA) *and Negative Affect*(NA) *Scales* (PANAS) [58] was administered to ensure that participants presented mood levels in the normal range. It consists of 20 items representing various mood characteristics (e.g., interested, distressed, excited, upset), for which the participants indicate how each characteristic corresponds to them on a 5-point Likert scale ranging from 1 (very little or not at all) to 5 (very much). Participants were told to rate their mood “at the moment” of completing the questionnaire. As there is, to our knowledge, no normative data of the PANAS for the French-Canadian general population (only for the French-Canadian athletes [57]), the normative data from the original validation study of the PANAS were used as reference (PA; *M* = 29.7, *SD* = 7.9; NA; *M* = 14.8, *SD* = 5.4) [58]. A Z score was calculated for each participant for the PA and the NA scales. All participants reported moods ranging into the normality, namely 3 standard deviations from the mean of normative group of reference [58].

#### Emotional valence and arousal scales

After each trial, participants were instructed to report their emotional experience in terms of valence using a 9-point SAM (self-assessment manikin) Likert-scale (1 = Very negative, 5 = Neutral, 9 = Very positive) [59]. Participants were also instructed to report their emotional experience in terms of arousal using a 9-point SAM Likert-scale (1 = Very low arousal, 9 = Very high arousal) [59]. These scales served three purposes: 1) to ensure that each participant perceived normal levels of valence compared to the group average; 2) to statistically control the differences in levels of arousal between the positive and negative social scenes; and 3) to maintain participants’ attention during the experimental task.

#### Situational empathy scale

For each trial, participants were also asked to report how empathic they felt for the target, using a visual analogue scale ranging from “not at all empathic—0” to “completely empathic—100”. By orienting the participant’s perspective toward the target, the cognitive component of empathy was postulated to be engaged to a greater extent. If participants asked for more clarification, the experimenter mentioned that they were to report their level of empathy by referring to their own conceptualization of empathy. The objective here, considering that empathy is a phenomenological and multidimensional concept, was to obtain a global measure of situational empathy reflecting the personal self-experience of the phenomenon.

Self-report measures were recorded using the *E-prime* 2.0 software. The average value for each self-report measure was calculated for each valenced social scene and emotion regulation condition. Trials corresponding to positive and negative social scenes in which self-reported valence diverged by more than 3 standard deviations from the average score of the group for each of the different videos, were considered outliers. This procedure led to the exclusion of 0.59% of all trials. The initial score for situational empathy corresponding to the final cursor position on the visual analogue scale was converted into a rating ranging from 0 to 100.

#### Dispositional empathy scale

In order to examine the relationship between situational and dispositional empathy, participants completed the Interpersonal Reactivity Index (IRI; [35, 36]) after the experiment. The IRI is a 28-item self-report questionnaire providing four subscales: Perspective Taking (PT), Fantasy (FS), Empathic Concern (EC), and Personal Distress (PD). This questionnaire measures different dispositional traits of empathy. The PT subscale provides a measure of the capacity to understand the perspective of others. The FS provides a measure of the tendency to identify oneself to characters in fictional situations. The EC subscale provides a measure of feeling compassion toward others in distress. The PD subscale provides a measure of personal distress when others experience negative emotions.

### Autonomic measures

All autonomic measures were acquired at 1000 Hz using a Biopac MP150 system using the AcqKnowledge software (AcqKnowledge 4.1, Biopac Systems Inc.). To synchronize each video stimuli with the autonomic signal, digital triggers were sent to the recording system and stored along with the autonomic data.

#### Heart rate variability analysis

Cardiac activity was recorded continuously with a standard three lead electrocardiogram (ECG) using a lead II configuration. Disposable electrodes with conductive hydrogel (ClothElectrodes, Repositionable, H59P, Medtronic) were used. One electrode was placed below the right clavicle (negative pole), one was placed on the left clavicle (ground), and one was placed on the lowest left rib (positive pole). An ECG100C Biopac module acquired the cardiac activity signal. The ECG data was processed using the open access HRV analysis software [60]. ECG Acknowledge files were imported in HRV analysis software.

The ECG signal was imported in the software at 1000 Hz to ensure good time resolution for the RRI [60]. The ECG signal was then denoised using wavelet transform passing through a moving averaging-based linear high-pass filter to highlight the QRS complex [61]. This automatic algorithm was developed by Pichot and colleagues [62]. It is based on a moving average-based method and has demonstrated a 99.5% detection rate in the MIT-BIH Arrythmia Database [62]. As recommended by established guidelines [16], each R peak was visually inspected for each participant. When the algorithm failed to detect a peak or provided an isolated artifact peak, the cubic-spline interpolated correction was applied. Therefore, for ectopic, missing, or false beats, the R peaks were manually corrected. A correction was needed for approximately 0.83% of the data across participants, which is in concordance with the detection rate ratio of the algorithm. Recording segments that were too noisy due to movement artifacts or loss of signal and that could not be rectified were discarded. This procedure led to 2.26% of discarded trials across participants.

In order to decompose the variance of the heart rate into its frequency components, continuous time wavelet transform analysis was used. These analyses were favored over classical Fourier transform analysis as wavelet transform allows the study of transient modifications in the RRI signal at any time and during very short periods of time. Wavelet transform analysis is also a valuable analysis tool to elucidate spectral information within the signal [19]. Wavelet transform analysis provides a means to overcome the poor frequency resolution of narrow time windows of the signal [19]. Briefly, a wavelet implies a small wave of finite duration and finite energy [61]. When using wavelet transform analysis, three levels, namely *levels 2*, *4*, and *8* corresponding respectively to different widths of the wavelet, are retained and compared to the RRI signal [62]. These levels covert the fast frequencies (0.15 to 0.4 Hz) in the RRI signal [60, 62] and correspond approximately to the Fourier high frequencies. The RRI signal is first re-sampled at 2.4 Hz [60]. The mother wavelet is then translated continuously along the time scale of the signal in order to evaluate wavelet coefficients at all instants of time [19]. By using the *Daubechies 4* mother function [60], the wavelet coefficients are calculated at each of the seven levels. The variability power is then calculated by the sum of the squares of the coefficients at each level [62]. The HF is then computed from the RRI signal for every second during forty seconds after the onset of each video for each trial. This led to forty HF measures for each trial. The average of the HF was then calculated for each trial. The average of the HF for each Valence and Emotion Regulation condition was calculated for each participant. HF data were transformed using a log transformation (log) to correct for skewness and kurtosis. For more information on wavelet transform to quantify heart rate variability, please see Pichot et al. [60, 62].

#### Respiration rate

Respiration amplitude and depth can influence HRV measures [16, 18]. Respiratory activity was thus gathered in order to control breathing and interpret HF data accurately [19]. Indeed, HF is influenced by breathing when the rates are between 0.15 Hz (9 cycles per minute) and 0.40 Hz (24 cycles per minute) [16, 19]. When respiratory rates remain between these rates, HF stays between the boundaries of those frequencies and thus, reflects vagal tone [16]. Respiratory activity was measured by the relative expansion changes of the thoracic abdominal, monitored by a pneumogram sensor, a self-inflating pressure pad, attached to a differential pressure transducer. The sensor was placed above the ECG electrode on the left ribcage using medical tape and the signal was recorded using a DA100C-TSD160B Biopac module.

Data were pre-processed and analyzed using Matlab R2015 8.5.0.197613. After importing the data, a low-pass filter with a cut off frequency of 1 Hz was applied. Inspiration peaks (maximum amplitude of each respiratory cycle) were first identified. A signal change of at least 0.075 mV was needed to qualify the beginning of a response. The maximum amplitude of that response was considered to be the inspiration peak. The time difference between two inspirations was then calculated and counted as one respiration cycle. As respiration rate is often used to index respiratory activity [63], this index was calculated. The mean respiration rate (i.e., number of breaths per minute (BPM)) at rest for a young adult man is 16 BPM and for a young adult woman is 19 BPM [64, 65]. There is, however, considerable inter- and intra-individual variation [66]. A range between 12 BPM (i.e. 6 sec for one respiration) and 20 BPM (i.e. 3 sec for one respiration) can be considered normal variations of the respiration rate at rest in human [63, 64, 66]. Based on these criteria, and in order to gather data regarding variations in respiration rate as related to different emotion conditions, the respiration rate range included was between 7.5 BPM (i.e. 8 sec for one respiration circle, namely the maximum time difference between two inspirations) and 60 BPM (i.e. 1 sec for one respiration circle, namely the minimum time difference between two inspirations). Time duration of a respiratory cycle can increase during an emotional task [67]. Respiration rates were finally calculated for each epoch of 40 seconds after the beginning of each trial. Respiration rates were then examined to ensure that they were within the analytic band of the HF. No respiration rates fell below the HF cut-off.

#### Electrodermal responses

Electrodermal activity was recorded using a GSR100C Biopac module. Disposable electrodes with isotonic gel (Biopac Systems, EL507) placed on both thenar and hypothenar eminences of palms of the non-dominant hand of the participants. Skin sites were washed beforehand with an alcohol pad. Electrodes were fixed with a medical ruban. Electrodermal signal was pre-processed and analyzed using Matlab R2015 8.5.0.197613. Criteria used to pre-process and calculate the EDRs were determined based on establish recommendations [20–22]. The signal was individually and visually inspected. Trials with loss of signal or trials in which the signal was too noisy to be corrected (due to recording or movements artifacts), were discarded. A 0.05 Hz high-pass filter was applied to eliminate skin conductance level variations related to tonic electrodermal activity and drift artifacts. As the EDR (EDRs-amp) has been shown to index local phasic increases of the sympathetic activity [20–22], this metric was calculated. EDRs-amp are expressed in MicroSiemens units (*μ*S). The minimum deflection criterion was fixed at 0.05 *μ*S, i.e., the signal had to increase by at least 0.05 *μ*S from the participant’s basic skin conductance level to be qualified as a new response [20, 21]. In the cases of superimposed responses, the evaluation method “B” was used, which has been used as standard [21]. More precisely, an EDR must be separated by at least 5 seconds from a preceding EDR and must increase again by at least 0.05 *μ*S, to be identified as new responses [20, 21]. The amplitude of each EDR was calculated for a time window of 40 seconds after the onset of the video presentation. The mean amplitude of all EDRs (EDRs-AmpMean) was calculated for each trial. The mean amplitude of all EDRs-AmpMean for each condition (EDRs-AmpMeantot) was then calculated for each participant. EDRs-AmpMeantot data were transformed using a log transformation (log + 1) to correct for skewness and kurtosis [68].

### Statistical analysis

Statistical analyses were performed with SPSS (Version 25.0; IBM Corp., Armonk, NY). The coefficients for the skewness and kurtosis of the distributions of the data for each dependant variable across each condition were first computed. These coefficients revealed that the distributions were not gaussian, but rather consistent with a gamma distribution. Generalized Estimating Equations (GEE) approach can manage this situation as a gamma distribution could be specified [69]. In addition, no specific covariance structure was able to be identified in our case. GEE allows for an unstructured covariance matrix between repeated measures. GEE also works efficiently with missing data without removing participants. Finally, the sample met an acceptable size of 50 clusters (participants) required for GEE analyses [70]. GEE analyses were thereby appropriate for the purposes of the current study.

A three-way GEE (proc GENLIN, distribution = Gamma, link = log, corrtype = unstructured) was conducted on situational empathy, RRI, HF, and EDRs. Emotion Regulation and Valence conditions were introduced as within-subject factors. Considering that the No-regulation condition was always presented first during the experiment, the block order was introduced as a covariate to control for testing order. Inherent post hoc pairwise t-tests comparisons from the GEE analysis procedure were computed for significant main and interaction effects. Differences were considered as significant when *p* < .05, after correction for multiple comparisons. A sequential Bonferroni correction was applied. The alpha was a two-sided threshold. Pearson correlations were used to examine the relationship between the score of situational empathy and the four subscales of the IRI for each of the conditions of emotion regulation and valence.

### Manipulation verification

To assess whether participants perceived the valenced social scenes as being either positive or negative compared to the neutral scenes, a GEE was performed on the valence self-report measures. Participants reported significantly different ratings of valence across the valence conditions [*Wald χ*^2^ = 504.418; *df* = 2, *p* < .001] and the emotion regulation conditions [*Wald χ*^2^ = 62.809; *df* = 2, *p* < .001]. Across conditions of emotion regulation, Negative Social Scenes were rated as being lower in valence (i.e., negatively valenced) compared to Neutral (*d* = 23.440, *df* = 1, *p* < .001) and Positive (*d* = 47.314, *df* = 1, *p* < .001) Social Scenes. Neutral compared to Positive Social Scenes were perceived as being lower in valence (i.e., neutral valence) (*d* = 28.178, *df* = 1, *p* < .001). The Up- compared to the Down- (*d* = 1.117, *df* = 1, *p* < .001) and No-regulation (*d* = 1.400, *df* = 1, *p* < .001) conditions were perceived as being more positively valenced. The Down- compared to the No-regulation (*d* = 4.936, *df* = 1, *p* < .001) conditions were perceived as being more positively valenced. However, the means for each emotion regulation condition all ranged between 3.442 and 4.755, which correspond to roughly a neutral valence (4/9 on the Likert scale). The main effect of emotion regulation was thereby rather small.

To assess if the valenced compared to the neutral social scenes were perceived as being similar in terms of arousal, a GEE was performed on the arousal self-report measure. Participants reported significantly different ratings of arousal across valence [*Wald χ*^2^ = 214.331; *df* = 24, *p* < .001] and emotion regulation conditions [*Wald χ*^2^ = 48.872; *df* = 2, *p* < .001]. Negative compared to Neutral Social Scenes were perceived as being more arousing (*d* = 9.124, *df* = 1, *p* < .001) and more arousing compared to Positive Social Scenes (*d* = 2.699, *df* = 1, *p* < .001). The Up- compared to the Down- (*d* = 6.368, *df* = 1, *p* < .001) and No-regulation (*d* = 2.552, *df* = 1, *p* < .001) conditions were perceived as being more arousing. The Down- compared to the No-regulation (*d* = 4.757, *df* = 1, *p* < .001) conditions were perceived as being less arousing.

## Results

### Effects of emotion regulation and valence on situational empathy

**Fig 1:**
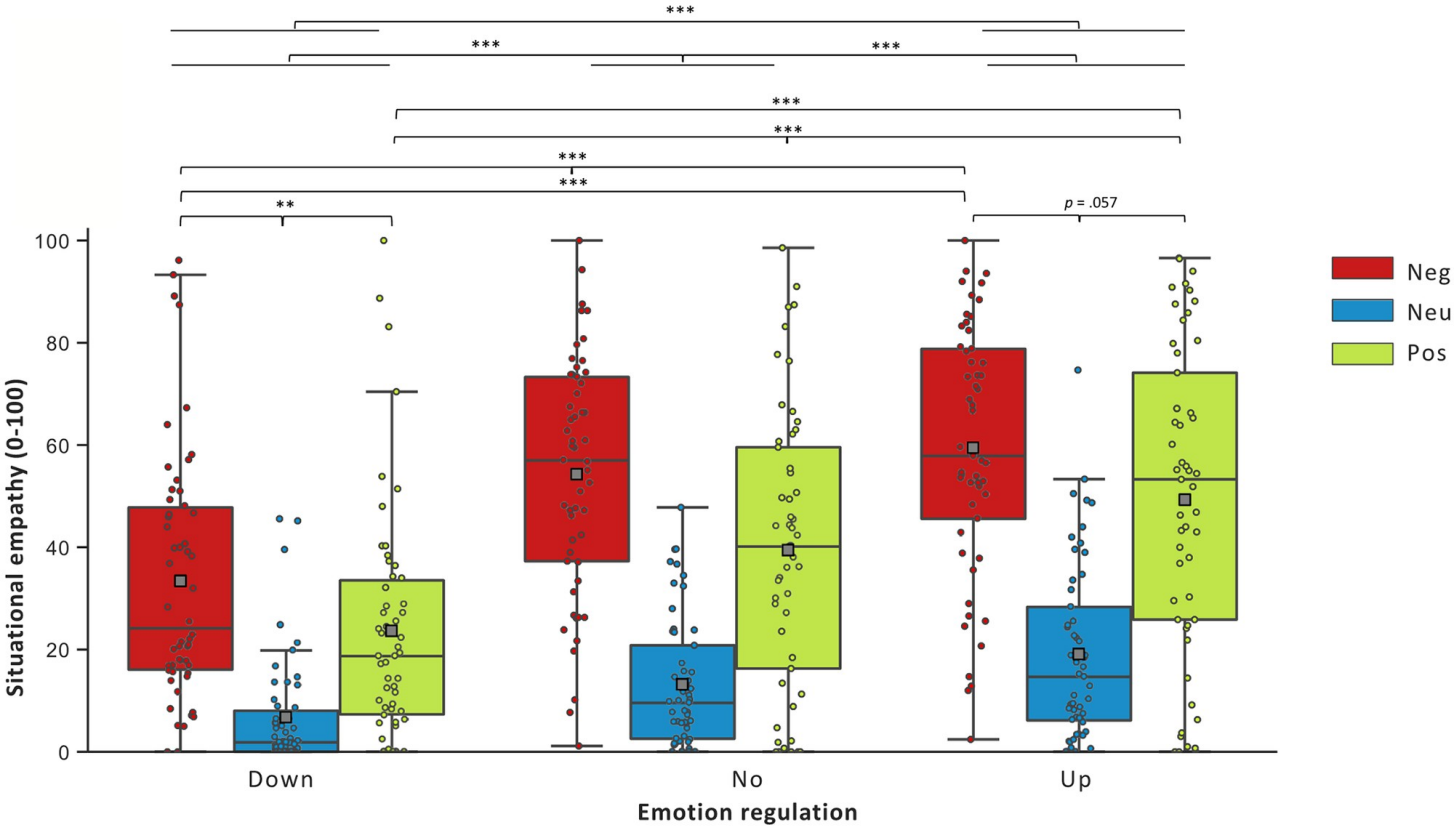
Mean of participants’ ratings on the situational empathy scale during emotion regulation and valence conditions. Abbreviations: M = Means, SE = Standard-Error, Down = Down-Regulation, No = No-Regulation, Up = Up-Regulation, Neg = Negative Social Scenes, Neu = Neutral Social Scenes, and Pos = Positive Social Scenes. *p < .05, **p < .01, ***p < .001.

A GEE showed a main effect of Emotion Regulation on situational empathy [*Wald χ*^2^ = 35.895; *df* = 2, *p* < .001]. Pairwise comparisons revealed that Up-regulation compared to No-regulation was related to greater situational empathy (*d* = 2.747, *df* = 1, *p* < .001). Up- compared to Down-regulation was related to greater situational empathy (*d* = 7, 678, *df* = 1, *p* < .001). No- compared to Down-regulation (*d* = 5.287, *df* = 1, *p* < .001) was related to greater situational empathy. A main effect of Valence was obtained on situational empathy [*Wald χ*^2^ = 140.643; *df* = 2, *p* < .001]. Negative compared to Positive Social Scenes (*d* = 3.043, *df* = 1, *p* < .001) were related with higher situational empathy. Negative (*d* = 15.161, *df* = 1, *p* < .001) and Positive (*d* = 11.190, *df* = 1, *p* < .001) compared to Neutral Social Scenes were related to higher situational empathy. A significant interaction effect was also found [*Wald χ*^2^ = 13.879; *df* = 2, *p* < .01]. During Up-regulation, Negative compared to Positive Social Scenes (*d* = 2.134, *df* = 1, *p* = .057) were related to higher situational empathy. During Down-regulation, Negative compared to Positive Social Scenes (*d* = 2.434, *df* = 1, *p* < .01) were related to higher situational empathy. See Fig 1.

In order to examine if differences in self-reported arousal between Positive and Negative Social Scenes might explain the main effect of Valence on situational empathy, a secondary set of analysis was conducted in which the self-report measure of arousal was introduced as a covariate. This GEE yielded the same pattern of findings reported above. Thus, the main effect of Valence on situational empathy was not attributed to the arousal as perceived by the participants.

### Effect of emotion regulation on autonomic responses

**Fig 2:**
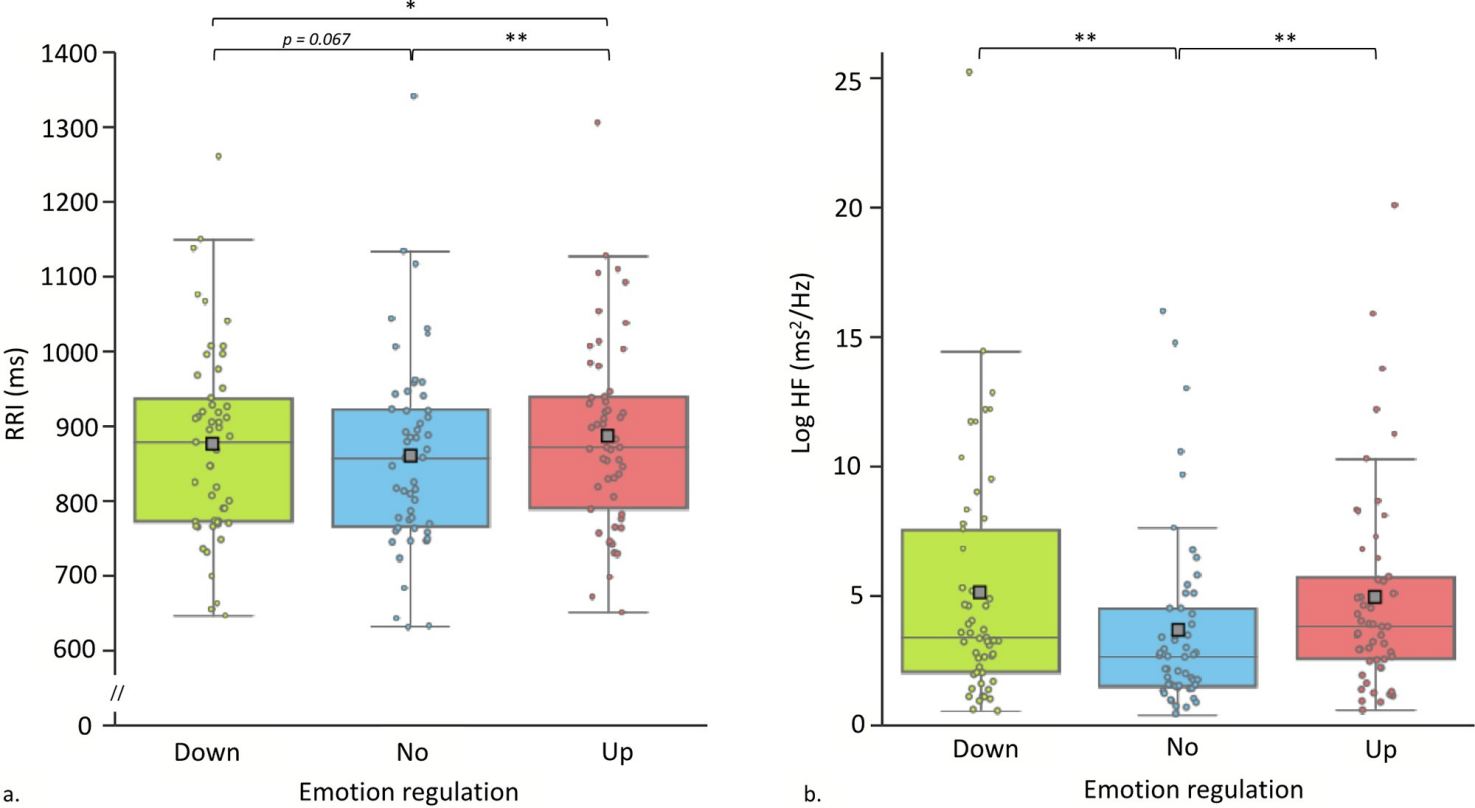
Participants’ average cardiac indexes during emotion regulation conditions. Abbreviations: RRI = RR intervals, Log HF = log-transformed of high frequency, ms = milliseconds, Down = Down-regulation, No = No-regulation, Up = Up-regulation. *p < .05, **p < .01.

**Fig 3:**
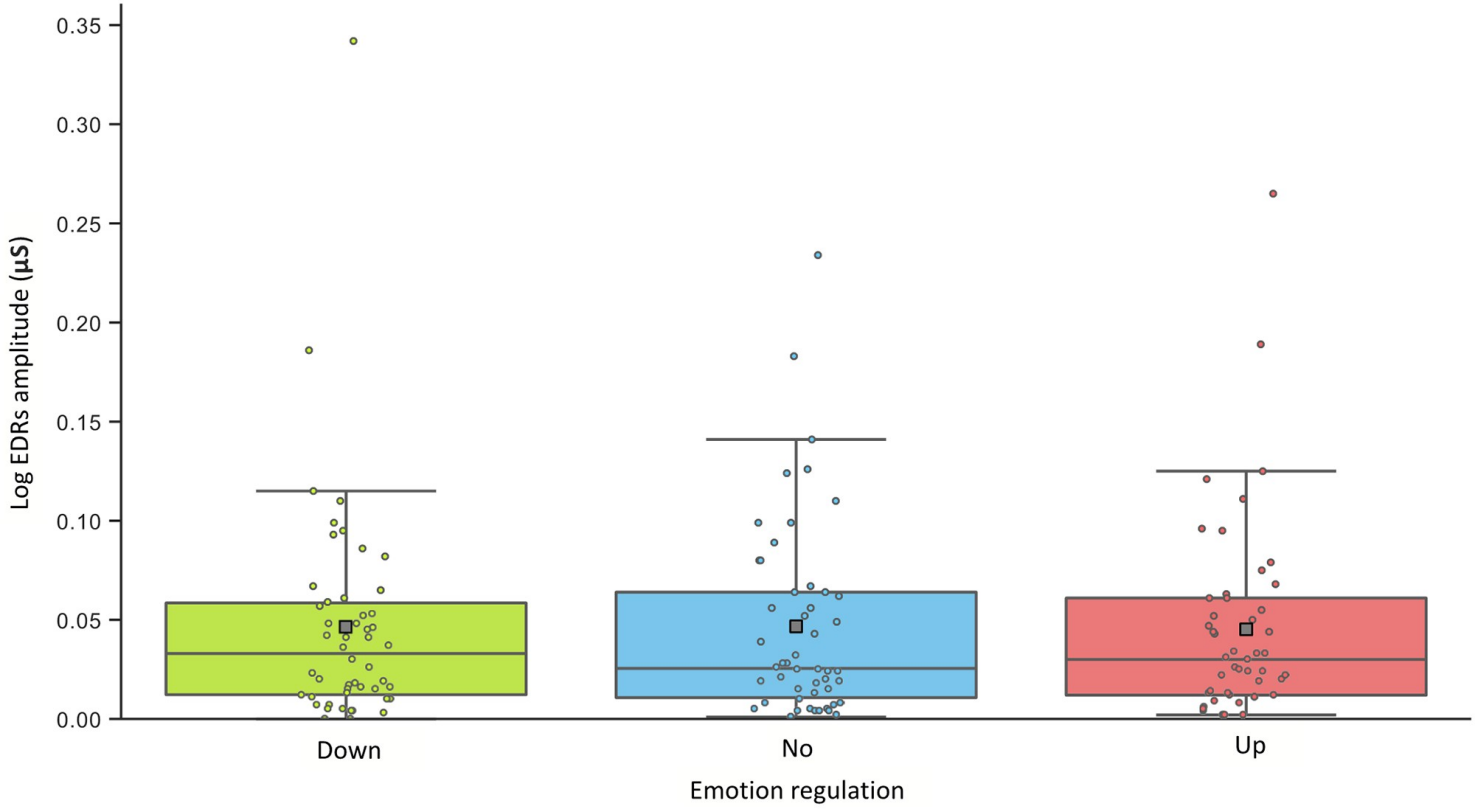
Participants’ average electrodermal responses during emotion regulation conditions. Abbreviations: Log EDR AmpMeantot = log-transformed of the mean of the electrodermal phasic responses, ms = milliseconds, Down = Down-regulation, No = No-regulation, Up = Up-regulation.

The GEE on RRI revealed a significant main effect of Emotion Regulation [*Wald χ*^2^ = 9.557; *df* = 2, *p* < .01]. An increase of the RRI was observed during Up- and Down- compared to No-regulation (Up > No: *d* = 0.866, *df* = 1, *p* < .01; Down > No: *d* = 0.437, *df* = 1, *p* = .067). Up- compared to Down-regulation was also related with an increase of the RRI (*d* = 0.430, *df* = 1, *p* = 0.05). See Fig 2a. The GEE on HF revealed a significant main effect of Emotion Regulation [*Wald χ*^2^ = 11.571; *df* = 2, *p* < .01]. An increase was observed during Up- and Down- compared to No-regulation (Up > No: *d* = 0.658, *df* = 1, *p* < .01; Down > No: *d* = 0.430, *df* = 1, *p* < .01; Up = Down, n.s.). See Fig 2b. The analysis on EDRs-AmpMeantot [*Wald χ*^2^ = 0.200; *df* = 2, *p* < .905] did not showed any significant difference. See Fig 3.

### Effect of valence on autonomic responses

**Fig 4:**
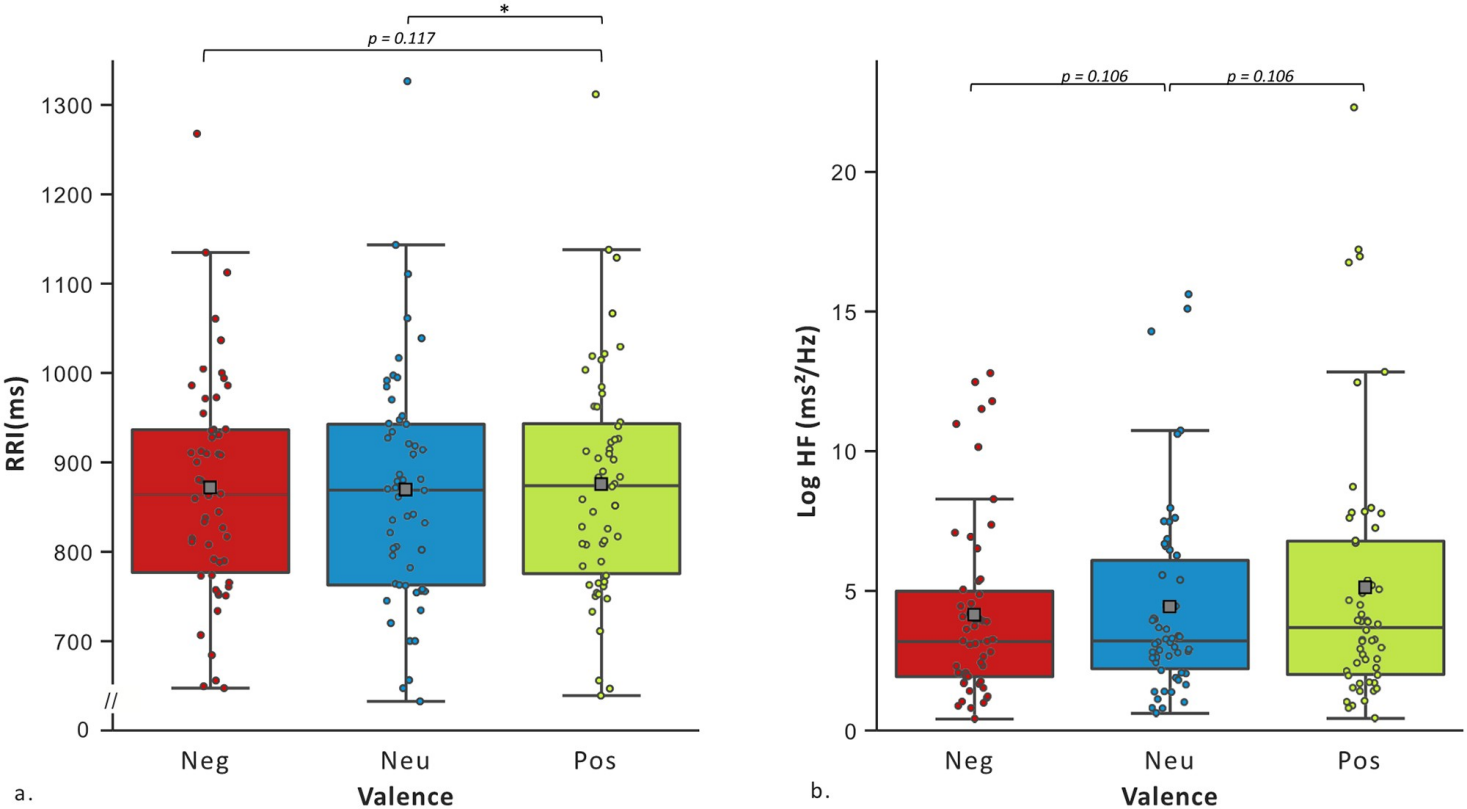
Participants’ average cardiac indexes during valence conditions. Abbreviations: RRI = RR intervals, Log HF = log-transformed of high frequency, ms = milliseconds, Neg = Negative Social Scenes and Pos = Positive Social Scenes. *p < .05.

**Fig 5:**
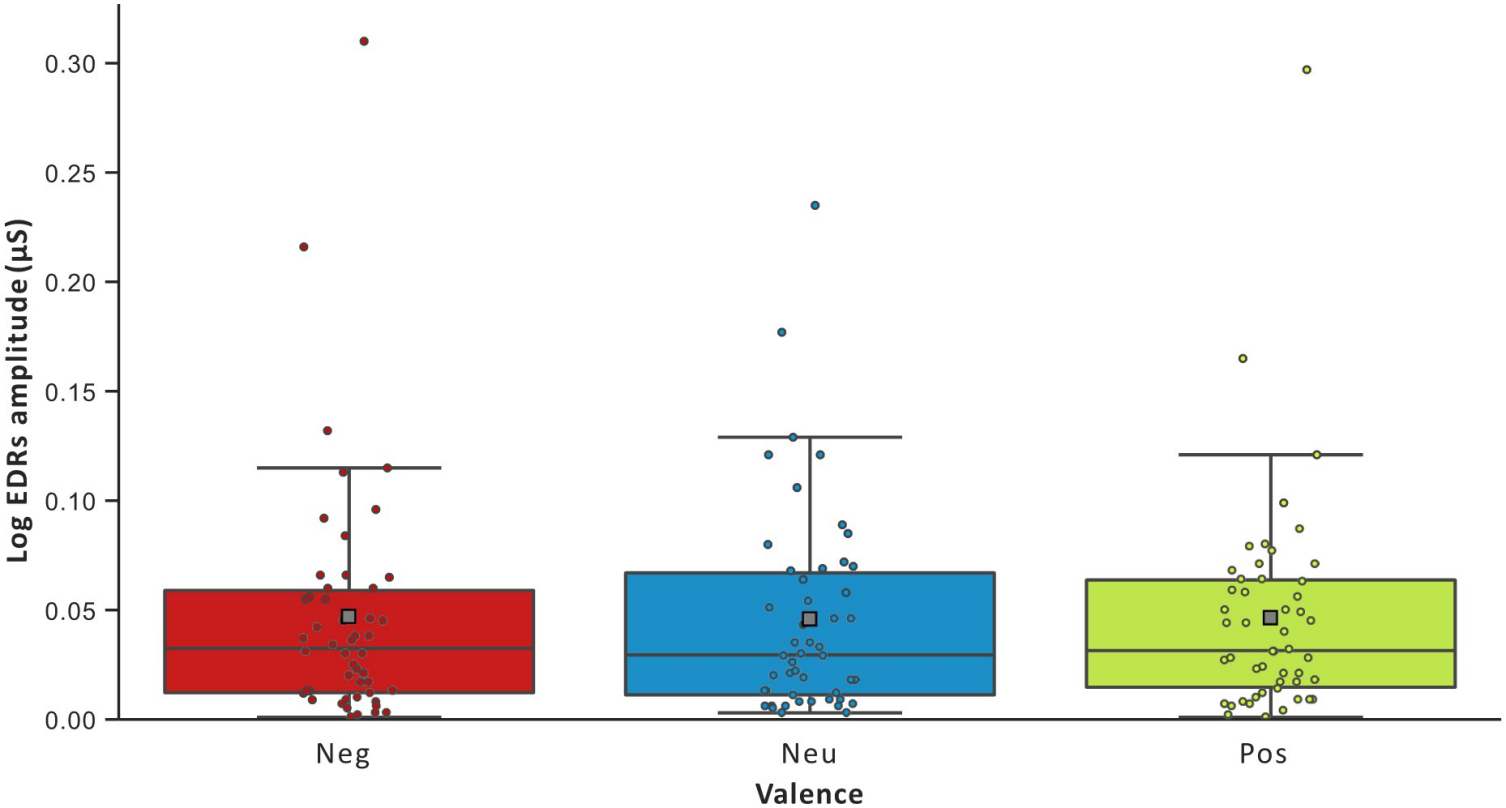
Participants’ average electrodermal responses during emotion regulation conditions. Abbreviations: Log EDR AmpMeantot = log-transformed of the mean of the electrodermal phasic responses, ms = milliseconds, Neg = Negative Social Scenes and Pos = Positive Social Scenes.

The GEE on RRI revealed a significant main effect of Valence [*Wald χ*^2^ = 8.281; *df* = 2, *p* < .05]. Positive compared to Neutral (*d* = 0.228, *df* = 1, *p* = .05) Social Scenes were related to an increase of the RRI, and were slightly higher compared to Negative (*d* = 0.156, *df* = 1, *p* = .117) Social Scenes. See Fig 4a. The GEE on the HF revealed a significant main effect of Valence [*Wald χ*^2^ = 6.130; *df* = 2, *p* < .05]. Positive (*d* = 0.242, *df* = 1, *p* = .106) and Negative (*d* = 0.279, *df* = 1, *p* = .106) compared to Neutral Social Scenes were related to an increase of HF. See Fig 4b. The analysis for the EDRs-AmpMeantot did not showed any significant difference [*Wald χ*^2^ = 0.271; *df* = 2, *p* < .873]. See Fig 5.

### Interaction effect of emotion regulation and valence on autonomic responses

No interaction effect between Emotion Regulation and Valence was observed on RRI [*Wald χ*^2^ = 2.123; *df* = 4, *p* < .713], HF [*Wald χ*^2^ = 3.893; *df* = 4, *p* < .421], or on EDRs-AmpMeantot [*Wald χ*^2^ = 1.089; *df* = 4, *p* < .896].

### Relationship between situational and dispositional empathy

Significant correlations were found between situational empathy ratings during some Emotion regulation conditions and dispositional empathic concern. Positive correlations were found between the situational empathy score and the EC score of the IRI during the No-regulation condition for Negative Social Scenes [*r*(52) = 0.27, *p* = .03] and Positive Social Scenes [*r*(52) = 0.24, *p* = .05]. Positive correlations were also found between the situational empathy score and the EC score of the IRI during the Down-regulation condition for Negative Social Scenes [*r*(53) = 0.29, *p* = .02] and Positive Social Scenes [*r*(53) = 0.24, *p* = .04]. No significant correlations were found between the situational empathy score and the EC score of the IRI during the Up-regulation condition for Negative Social Scenes [*r*(53) = 0.18, *p* = .10] and Positive Social Scenes [*r*(52) = 0.17, *p* = .11]. Considering these results, and in order to test the relationship between participants’ dispositional empathy and emotion regulation capacities, additional analyses were conducted. The differences of scores on the situational empathy scale between the No-regulation and Down-regulation conditions (i.e., No-regulation minus Down-regulation on the situational empathy scores) for Positive and Negative Social Scenes were calculated separately. The correlations were not significant between these differences in situational empathy scores and the scores on the EC scale for Positive Social Scenes [*r*(52) = 0.06, *p* = .35] and Negative Social Scenes [*r*(52) = 0.03, *p* = .43].

## Discussion

Emotion regulation has been proposed to be a component of empathy [1, 3, 9]. However, experimental data supporting the effect of emotion regulation on empathy and its related autonomic activation remains scarce. The current study served to deepen our understanding of this issue by assessing the effect of emotion regulation on situational empathy and metrics of sympathetic and parasympathetic activation. This study also provides initial data comparing the effect of viewing positively and negatively valenced social scenes on situational empathy and autonomic responses. To our knowledge, no study has previously addressed these empirical questions, which are essential to broaden our understanding of how emotion regulation and empathy are related at the experiential and autonomic level. Our results showed that up- and down-regulation were associated respectively with an increase and decrease of situational empathy, in parallel with an increase of autonomic activation (i.e., HF component), mainly mediated by the parasympathetic branch of the ANS. These conditions did not lead to increased activation of our measure of the sympathetic branch (i.e., EDRs). Additionally, positive compared to negative socio-emotional scenes were related with a slight increase of the HRV (i.e., RRI), in addition to reduced situational empathy. Results will be discussed in detail with regard to previous literature. Strengths and limitations will be presented.

### Emotion regulation impacts situational empathy

Results of the study can be related to Eisenberg’s work examining the link between emotion regulation and empathy. In Eisenberg and colleagues’ view, individuals tended to experience either personal distress or empathic concern in responses to others’ needs, which vary according to individual differences in emotion regulation capacities [11, 71]. Individuals with high emotion regulation capacities are more prone to empathic concern (or sympathy), as they can modulate their vicarious emotions to maintain an optimal level of arousal. Conversely, individuals presenting with lower emotion regulation capacities are more prone to personal distress, as they struggle to keep high arousal in an optimal range. Findings of the current work support some points of Eisenberg’s view of empathy. Indeed, we found significant positive correlations between dispositional empathic concern and situational empathy for positive and negative social scenes during no- and down-regulation. Results also showed that down-regulation decreased situational empathy and up-regulation increased situational empathy compared to the control condition. These empirical data provide support for Eisenberg’s idea that cognitively engaging emotion regulation processes modulates situational empathic responses.

One the other hand, up-regulation, namely, to feel the another’s feelings as oneself, was related to greater situational empathy and did not correlate with more dispositional empathic concern or personal distress. Down-regulation, namely, to view others’ feelings from a distanced/third-person perspective, was related to less situational empathy. This pattern of responses is less consistent with Eisenberg’s hypotheses and is also less consistent with other models [1, 3]. These results also diverge from the initial hypothesis. Indeed, it was expected that up-regulation would be associated with a decrease in situational empathy, while down-regulation would be associated with an increase in situational empathy. One possible explanation may be due to the level of emotional intensity depicted by the films. Indeed, participants perceived the social emotional films as being moderate in terms of arousal. At this level of arousal, it is possible that up-regulating emotions had the effect of increasing participants’ levels of arousal into a range that engaged higher situational empathy without being aversive to oneself. Decety and Lamm [2] suggested that up-regulation is important to promote empathic response towards others, especially when others’ emotions do not engage oneself in an emotional state. Another possible explanation could be related to the nature of the emotions depicted during the video clips. Indeed, most models, and the supporting literature on empathy, have been built around the specific case of empathy for others in physical pain. Here, the negative social scenes illustrated a variety of emotions, including pain, but also sadness and anger. Perhaps, these emotions, when expressed by others, promoted different reactions, such as avoidance, among the participants as they viewed the scenes. In this case, the need to explicitly try to put oneself in another’s shoes (such as in the up-regulation condition) might be necessarily to enhance empathy. As the nature of the empathic responses, i.e. what emotions the participants were feeling, while viewing the films was not documented, further investigations are needed to shed light on the question. Finally, these findings suggest that the effect of emotion regulation on situational empathy might not necessarily be related to participants’ dispositional empathic capacities. Perhaps, dispositional empathy is not a clear indicator of how empathic participants’ will be in a specific situation and is even a less clear indicator when emotion regulation capacities comes into play.

Linking Batson’s work to the current findings also leads to interesting interpretations. Indeed, Batson’s studies demonstrated that different perspectives can influence situational empathy. In Batson, Early, and Salvarani’s study [72], different instructions were given to participants while they were listening to a radio interview of a person in serious need. In the objective-perspective condition, participants were to be objective and detached from the person’s feelings. In the imagine-self condition, participants were to imagine how they would feel in the situation. In an imagine-other condition, participants were to imagine the person’s feelings. Results showed that imagine-other and imagine-self compared to the objective-perspective condition were related to more empathy and altruistic motivation. Imagine-self was related to more personal distress than imagine-other or the objective-perspective. In two other experiments, Batson and colleagues [73] showed that the effect of different types of perspectives-taking depends on the type of task performed by the participants. In a task-assignment paradigm, participants were instructed to assign either oneself or another person to a more desirable task or to a neutral task. Imagining oneself in the other’s situation did not increase moral action (i.e. assigning the other to the desirable task) compared to a no-perspective condition. Imagining the other’s feelings did increase empathy and moral action compared to the self-oriented perspective and no-perspective condition. In a second experiment, participants were instructed to either accept an initial task that would give themselves highly positive consequences and the other no positive consequences, or to accept a task that would give themselves and the other person moderately positive consequences. Imaging oneself in the other’s place compared to a no-perspective condition did increase moral action and empathy. In brief, imagining the other’s feelings is more effective at increasing empathy, altruistic motivation, and moral action. Putting oneself in the other’s shoes increases empathy, but also personal distress and egocentric motivation.

Some of the current findings are in line with Batson’s studies. During the up-regulation condition, namely when participants needed to feel another person’s feelings as their own, situational empathy increased. This result is consistent with Batson’s studies [72, 73] and particularly with the imagine-self condition. Our results might suggest that when participants are up-regulating, personal distress might also be higher. However, as no significant correlations were found between the scores on the Personal Distress subscale of the IRI during the Up-regulation conditions (Positive and Negative Social Scenes) in our study, this hypothesis is less plausible. During the down-regulation condition, namely when participants needed to make a “distance with other’s emotions”, situational empathy decreased. This is also consistent with the result in Batson’s study [72] showing that an objective perspective is related to reduced empathy. Taken together, it seems that taking a more objective or distanced perspective toward the other’s feelings may result in feeling less empathy for the other.

Other results were, however, inconsistent with Batson’s previous work. In Batson’s series of studies [73], the imagine-self condition did not lead to greater empathy during the task-assignment paradigm, but it did lead to greater empathy during the moral dilemma of fairness task. In the current study, up-regulation was related to greater empathy. Perhaps the divergence of results stems from paradigm differences. In Batson’s studies, different perspectives were examined based on paradigms imposing a decision that involved a certain form of competition of resources between oneself and the other (i.e., assigning oneself or the other to a desirable task, assigning oneself or the other to a task offering highly positive consequences). In this context, it might be harder for participants to engage themselves in an empathic response. In the current study, the experimental task was designed without any form of competition of resources or decision to make (other than indicating the level of situational empathy felt toward the target). Perhaps, in this context, an empathic response might be easier to engage. The physiological results seem to support this hypothesis, as the up-regulation condition compared to the no-regulation was not significantly related to greater sympathetic activity (no greater electro-dermal responses), but rather greater parasympathetic activation (greater HF power).

### Emotion regulation increased parasympathetic activity during situational empathy

Results revealed that down- and up-regulation were associated with an increase of the RRI and the HF compared to a control condition without any change of the EDRs-Amp. This pattern suggests that emotion regulation conditions were related with an increase of autonomic activity that was predominantly under the control of the parasympathetic branch. In addition, these findings were related with differences in situational empathy responses. The initial hypotheses were thus partly confirmed, but results are still consistent with previous literature. As mentioned earlier, emotion regulation is usually related with an increase of the HRV metrics. Participants were thus likely engaged their parasympathetic system in order to adequately down-regulate their emotions and engage themselves in an adapted empathic response. These results are also in line with Stellar and colleagues’ [37] study suggesting that compassion is related with an increase of parasympathetic activity and with Lischke and colleagues’ [32] study showing that higher dispositional empathy is related with higher HF (parasympathetically mediated).

Results are also in line with the neurovisceral integration model [25] and previous neuroimaging studies that link HRV to emotion regulation [24]. The main assumption of this model is that higher vagal tone is related with greater executive cognitive performance, as well as emotion regulation. Research on reappraisal consistently reports activation in cerebral structures related to evaluation and regulation/control systems, underpinned notably by lateral, medial, and cingulate prefrontal cortices and subcortical regions, such as the amygdala and the striatum [74]. A study using transcranial direct-current stimulation showed that enhancing the activity of the dorso-lateral prefrontal cortex improved participants’ capacities to down-regulate and up-regulate negative emotions, as measured by a decrease and an increase in arousal ratings, respectively, and by skin conductance responses [75]. In line with the current study, it seems that situational empathy is a flexible phenomenon for which up-regulation and down-regulation would involve regulatory mechanisms that are primarily parasympathetically mediated. These results also suggest that situational empathy can be modulated though the used of cognitively mediated top-down processes. One can argue that results obtained between emotion regulation conditions and the control condition might be related to mental effort/load. Most studies on the effects of cognitive load showed a reduction of HF bands [76], for instance during mental arithmetic tasks [77, 78], reading and talking [77], and attention/execution [79] and memory tasks [80]. Results from the current study might suggest that prefrontal cortices related to emotion regulation are involved during cardiac vagal control which in turn modulate empathic responses. Finally, the concomitance of experiential and autonomic results is particularly interesting, as it suggests that participants’ responses on the situational empathy scale were not exclusively related to social desirability and may possibly be related to generating adequate empathic responses.

### Emotion regulation of others’ positive vs. negative emotions is related to lesser situational empathy and greater parasympathetic influence

The ability to feel and understand others’ distress is fundamental to human social interactions [1]. However, the ability to share others’ joy might not be as automatic and/or may serve distinctive social functions. Results of the current study showed distinct patterns of experiential and cardiac regulated responses during empathy for others’ positive compared to negative emotions. Specifically, results showed that processing others’ positive compared to negative emotions was associated with reduced situational empathy and an increase of the HRV (i.e., RRI). Although most of HRV metrics can be primarily related to the parasympathetic influence, RRI is also related to a relative influence of the sympathetic branch [16]. A review on ANS activity during self-processing of emotions reported different patterns of peripheral responses related with emotional valence: positive and negative emotions were both characterized by an increase of electrodermal and respiratory activity [81]. Various positive and negative emotions differed, however, in sympathetic/parasympathetic cardiac responses. Positive emotions, such as affection, amusement, and joy were associated with increased parasympathetic influence, while happiness was associated with increased sympathetic activation. In contrast, negative emotions, such as anger, anxiety, fear, or crying sadness were characterized by an increase of cardiac sympathetic activation. An increase of cardiac vagal control (parasympathetic activity) was observed during non-crying sadness [81]. Globally, positive emotions are more related to an increase in parasympathetic influence, whereas negative emotions are more associated with an increase in sympathetic influence.

Results of the current study suggest that this pattern of regulated cardiac response is, at least in part, reversed. From an evolutionist perspective, it has been suggested that empathy developed in humans to protect ourselves from danger, as well as to ensure the survival of others, if needed [3]. In this perspective, it is not surprising to find that others’ negative emotions elicit more situational empathy compared to positive ones. In order to prevent emotional contagion and adequately empathize with others, others’ negative emotions may need to be experienced with a relatively lower emotional intensity, which could explain the lower level of autonomic activity found in the current study. However, another interpretation is also possible. Although empathy for positive emotions is generally related to greater well-being [4], viewing someone else in a positive emotional state can also signal that this person is in an advantageous situation compared to oneself, thus enhancing negative emotions such as jealousy. This phenomenon is known has the *Glückschmerz* effect and has been demonstrated in the context of inter-group competition [82, 83]. Cikara and colleagues [82] showed that viewing the success of out-group members is associated with increased activation in the insula and the anterior cingulate cortex, brain structures activated by vicarious pain [84] and negative emotions [85]. Given that many of the positive scenes in the current study illustrated loving couples in happy situations, for some participants, this may have triggered memories related to their own romantic situations. For some participants, empathizing with others’ positive emotions may have been more difficult, and may have enhanced emotions of competition, jealousy, or even sadness, which may have engaged greater sympathetic activation and led to lesser situational empathy. In order to validate this hypothesis, it would have been interesting to assess participants’ feelings toward each video by adding self-report scales of discrete emotional feelings after each video or after the experiment. Future investigations on the effect of individuals’ differences in envy and/or jealousy on empathy for positive emotions during in-group versus out-group contexts could also provide important insights on fundamental aspects of empathy.

### Strengths and limitations

This study presents some strengths that are worth mentioning. The psychophysiological approach presented here, namely the combination of self-report and autonomic measures provides a more comprehensive understanding of empathy and helps to overcome the methodological limitations of both types of measures [12]. On one hand, self-report measures are relevant in order to obtain a more holistic and experiential measure of empathy. However, self-report measures might lack of objectivity. Studies have pointed out inconsistencies between self-report measures of empathy and have identified a weak inter-correlation between them [86]. It is therefore essential to corroborate self-report measures with more objective measures, which permit the quantification of other aspects of the phenomenon of empathy. On the other hand, although more objective, the interpretative links that can be drawn between physiological measures and the concept of empathy are rather difficult, as physiological measures provide specific information related to some autonomic mechanisms. A psychophysiological approach overcomes these respective limits by allowing the subjective experience of empathy to be linked to indicators of change of the peripheral nervous system [12].

This study has limitations that should also be considered. First, the video used in the current study were based on validation studies [50–52] conducted on French, Portuguese, and Spanish European populations and were extracted from films of different cultural backgrounds (French, Italian, British, and US cinema). As the current study was conducted on a French-speaking Canadian population, a cultural bias may have influenced the results. In order to address this issue, further research will be needed to expand the results of the current study by using validated extracts of films of the French-Canadian cultural background. Second, from a theoretical and methodological perspective, the current study only investigated one type of emotion regulation strategy, namely reappraisal. This restricts the scope of the findings to other types of emotional regulation processes, such as suppression or distraction. Further studies will be needed to investigate other types of emotional regulation strategies. Finally, the interpretation of self-report measures of situational empathy is prone to some bias. Indeed, participants needed to respond on the scale based on their own definition of empathy, which can bring greater variability in what exactly this scale was measuring. As empathy is a difficult construct to conceptualize [87], it cannot be excluded that participants were reporting other related psychological processes, such as personal distress, sympathy, or compassion. The development of more robust scales of situational empathy measures in future research could certainly enhance our comprehension on which and how components of empathy can be modulated by emotion regulation.

## Conclusion

The links established here between emotion regulation and empathy from an experiential and autonomic perspective are critical as they allow an in-depth understanding the processes involved in empathy. It has been demonstrated that emotion regulation engaged cardiac vagal control, which was associated with changes in situational empathy, possibly in order to adapt the empathic response.

Additionally, the current study provides empirical evidence suggesting fundamental differences between situational empathy for others’ positive and negative emotions. Furthermore, the present study relied on self-reports and physiological measurement to assess emotion regulation in empathy. Given that many studies have shown links between brain and autonomic activation [24] and between brain activation and empathy [84], future research combining self-report, cardiac, and brain measurements is needed to study emotion regulation in empathy.

## Supporting information

S1 Appendix Additional information regarding the videos used. (DOCX)
