# Supplementary material for: Emotion regulation of others’ positive and negative emotions is related to distinct patterns of heart rate variability and situational empathy
Source: PLoS One. 2020 Dec 31;15(12):e0244427. doi: 10.1371/journal.pone.0244427 (PMC7774949; doi:10.1371/journal.pone.0244427)
Supplement: S1 Appendix — (DOCX) [file pone.0244427.s001.docx]

**S1 Appendix. Additional information regarding the videos used.**

The targets in the videos were individuals from all ages, mostly middle-aged adults, but the sample also included two videos with a boy and one with a senior man. The number of women and men was roughly equally distributed across the social scenes. More precisely, amongst the negative social scenes, there were three middle-aged women and five middle-aged men, one boy, and one senior man. Amongst the positive social scenes, there were seven women and six men, all middle-aged adults. Amongst neutral social scenes, there were four middle-aged women and five middle-aged men and one boy.
